# Les maladies transmises par les moustiques en République de Djibouti: une revue de la littérature

**DOI:** 10.48327/mtsi.v5i4.2025.778

**Published:** 2025-11-10

**Authors:** Abdoulgabar ABDOURAHMAN OMAR, Oumnia HIMMI

**Affiliations:** 1. Centre de recherche en géophysique, patrimoine naturel et chimie verte (GEOPAC), Laboratoire géo-biodiversité et patrimoine naturel (GEOBIOL), Institut scientifique, Université Mohammed V de Rabat, Maroc.

**Keywords:** Maladies à transmission vectorielle, Paludisme, Filariose, Arboviroses, Revue de la littérature, Moustiques, République de Djibouti, Vector-borne diseases, Malaria, Filariasis, Arboviruses, Literature review, Mosquitoes, Republic of Djibouti

## Abstract

**Introduction - Justification:**

Cet article s’inscrit dans le cadre d’un projet doctoral visant à décrire l’écologie des moustiques présents sur le territoire de Djibouti et leur interaction avec les dynamiques épidémiologiques. S’appuyant sur une revue systématique de la littérature scientifique disponible, cette étude retrace l’évolution historique des pathologies transmises par les moustiques sur le territoire djiboutien.

**Matériel et méthodes:**

Cette étude a été conduite selon les normes PRISMA. Une recherche bibliographique exhaustive a été menée sur les maladies vectorielles transmises par les moustiques (arboviroses, parasitoses) dans le contexte géographique de l’actuelle République de Djibouti. PubMed, ScienceDirect, Springer Nature et Google Scholar ont été interrogées, afin de couvrir les publications indexées et la littérature grise. La syntaxe de requête combinait des opérateurs booléens (AND/OR) et des termes MeSH/Entrée pertinente, structurés en deux axes: « Territoire français des Afars et des Issas » et « Djibouti » avec « Dengue Djibouti », « Malaria Djibouti », etc.

**Résultats:**

À Djibouti, le risque d’infection par les arbovirus est principalement associé à des facteurs de risque environnementaux et comportementaux, avec un niveau plus élevé dans le centre-ville que sur le reste du territoire. La littérature scientifique recense au moins six maladies vectorielles transmises par les piqûres de moustiques femelles dont quatre font lobjet de cas documentés: le paludisme, le chikungunya, la dengue et la fièvre du Nil occidental. La fièvre de la vallée du Rift et le virus Zika sont également mentionnés, bien qu’aucun cas de ce dernier n’ait été officiellement signalé dans le pays à ce jour.

**Discussion - Conclusion:**

Les maladies transmises par les moustiques sont dominées par le paludisme et la dengue. Cela explique l’abondance des données sur ces deux maladies et leurs vecteurs. Des études sur la caractérisation des pathogènes et d’autres maladies transmises par les moustiques à Djibouti ainsi que sur la notification des signes cliniques sont fortement recommandées pour mieux comprendre les défis sanitaires.

## Abstract (en)

**Introduction -Justification.:**

This article is part of a doctoral project that aims to describe the ecology of the mosquitoes present in Djibouti and how they interact with epidemiological dynamics. This study traces the historical evolution of mosquito-borne diseases in Djibouti based on a systematic review of the available scientific literature.

**Materials and Methods:**

The study was conducted in accordance with PRISMA standards. An exhaustive bibliographic search was conducted on vector-borne diseases (arboviruses and parasitic diseases) transmitted by mosquitoes in the geographical context of the Republic of Djibouti. PubMed, ScienceDirect, Springer Nature, and Google Scholar were searched to cover both indexed publications and gray literature. The query syntax combined Boolean operators (AND/OR) and MeSH/relevant entry terms structured around two axes: “French Territory of the Afars and the Issas” and “Djibouti,” as well as “Dengue Djibouti,” “Malaria Djibouti,” etc.

**Results.:**

In Djibouti, the risk of arbovirus infection is primarily linked to environmental and behavioral risk factors, with a higher risk in urban areas than in rural regions. The scientific literature lists at least six vector-borne diseases transmitted by female mosquito bites. Four of these diseases have been documented in Djibouti: malaria, chikungunya, dengue, and West Nile fever. Rift Valley fever and Zika virus are also mentioned, though there have been no official reports of Zika virus cases in the country to date.

**Discussion -Conclusion.:**

Mosquito-borne diseases are dominated by malaria and dengue fever. This explains the abundance of data on these two diseases and their vectors. To better understand the health challenges, studies on the characterization of pathogens and other mosquito-borne diseases in Djibouti, as well as on the reporting of clinical signs, are strongly recommended.

## Introduction

Les moustiques sont connus pour leur capacité à inoculer des agents pathogènes, essentiellement des parasites et des virus. Ils sont à l’origine de maladies transmises aux humains et aux animaux [23]. Une revue récente de la littérature [2] mentionne 37 espèces de moustiques présentes à Djibouti, dont 8 sont des vecteurs potentiels de maladies telles que le paludisme, la filariose lymphatique, la dengue, la fièvre jaune, le virus du Nil occidental et le chikungunya. Il faut noter l’introduction *d’Anopheles stephensi* en 2012 en lien avec l’augmentation des cas de paludisme dans le pays [25].

**Figure 1:**
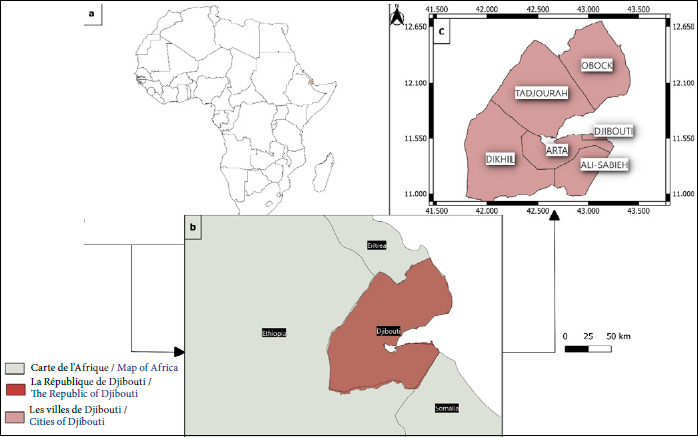
Carte de la République de Djibouti: (a) situation en Afrique, (b) pays frontaliers et (c) régions

La République de Djibouti est une ancienne colonie française située dans la corne de l’Afrique. Le pays est bordé par l’Érythrée au nord, l’Éthiopie à l’ouest, la Somalie au sud et la mer Rouge à l’est (Fig. 1).

Ce travail s’inscrit dans le cadre d’un projet de recherche doctoral sur l’écologie des moustiques à Djibouti et fait suite à la publication d’une revue de la littérature consacrée aux Culicidae dans ce pays [2]. Son objectif est de proposer une synthèse claire et exhaustive des maladies transmises par les moustiques et les agents pathogènes associés, en s’appuyant sur les références disponibles.

## Matériel et méthodes

Conformément aux lignes directrices PRISMA *(Preferred Reporting Items for Systematic Reviews and Meta-Analyses),* une revue de la littérature publiée sur les maladies transmises par les moustiques à Djibouti a été réalisée. Les bases de données interrogées sont PubMed, Science Direct, Google Scholar et Springer Nature. Afin de maximiser l’exhaustivité de la recherche et de limiter les biais de sélection, les mots-clés ont été utilisés en anglais et en français, couvrant les principales maladies vectorielles concernées. Les termes de recherche employés étaient les suivants:

- « *Moustiques de Djibouti* » / « *Djibouti mosquitoes* »;
- « *Maladies vectorielles à Djibouti* » / « *Vector-borne diseases in Djibouti* »;
- « *Dengue Djibouti* »;
- « *Fièvre jaune Djibouti »* / « *Yellow Fever Djibouti* »;
- « *Fièvre de la vallée du Rift Djibouti* » / « *Rift Valley Fever Djibouti* »;
- « *Filariose lymphatique Djibouti* » / « *Lymphatic filariasis Djibouti* »;
- « *Virus du Nil occidental Djibouti* » / « *West Nile virus Djibouti* »;
- « *Paludisme Djibouti* » / « *Malaria Djibouti* »;
- « *Zika Djibouti* »;
- « *Encéphalite japonaise Djibouti* » / « *Japanese encephalitis Djibouti* ».

Les études incluses dans cette revue sont toutes des études de recherche originales. Elles décrivent les résultats sur les maladies transmises par les moustiques dans la République de Djibouti notamment sur la diversité moléculaire et génétique, les facteurs de risque humains et animaux associés aux conditions écologiques et climatiques, ainsi que la modélisation spatio-temporelle et prédictive du risque.

**Figure 2:**
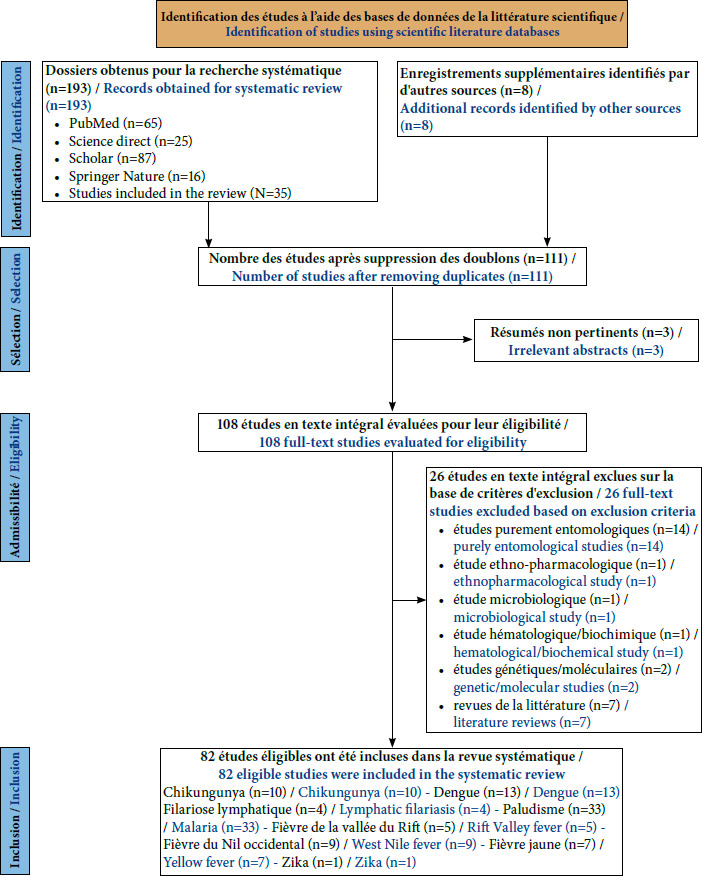
Organigramme de la sélection d’articles pour une revue systématique des maladies transmises par les moustiques à Djibouti

Nous avons exclu les articles mentionnant les maladies transmises par les moustiques mais portant davantage sur des études purement disciplinaires, en lien avec la microbiologie, l’entomologie et l’hématologie, voire des articles de revue comme l’illustre la Figure 2.

## Résultats

Au total, 193 articles ont été identifiés dans le cadre de cette revue systématique de la littérature portant sur les maladies transmises par les moustiques en République de Djibouti. Après élimination des doublons et des articles non pertinents, 82 références ont été retenues pour analyse (Fig. 2).

Le présent article propose une synthèse des principales pathologies arbovirales et parasitaires d’importance épidémiologique majeure sur le territoire djiboutien.

### Le paludisme

Djibouti présente une transmission palustre instable [1,64]. Après des premiers cas signalés en 1901 [10,11,57], l’endémie s’est installée en 1991 avec des pics à 7 000 cas/an [54,78]. Deux épidémies urbaines (2013-2014) ont conduit à 70 000 cas en 2020, liées à *Anopheles stephensi* [25,85,92]. L’analyse des cas confirmés révèle une prédominance nette de *Plasmodium falciparum,* responsable d’environ 90% des infections [39]. Les données récentes (2018-2021) confirment cette tendance avec 83% des cas attribués à cette espèce [59]. *Plasmodium vivax* représente quant à lui 5 à 10% des cas globaux [39], avec une proportion de 11% durant la période 2018-2021 [59]. *Plasmodium ovale* reste marginal, ne concernant que 3% des infections [72]. La transmission persiste toute l’année mais présente une saisonnalité marquée, avec une intensification entre novembre et mai [88]. Les études génétiques montrent une évolution de la diversité parasitaire, élevée avant 1999 [79], puis réduite par la suite [7], reflétant probablement des modifications dans les dynamiques de transmission et l’influence des importations du parasite depuis l’Éthiopie voisine [28,38].

L’arrivée *dAnopheles stephensi* en 2012 a profondément modifié l’épidémiologie du paludisme à Djibouti [25,85]. Ce vecteur présente plusieurs particularités inquiétantes: une résistance aux quatre principales classes d’insecticides [85], une excellente adaptation aux environnements urbains et un comportement endophile marqué. *Anopheles gambiae* et *Anopheles arabiensis,* vecteurs traditionnels, jouent désormais un rôle secondaire [92]. La lutte contre ces vecteurs est rendue plus difficile par la faible couverture en moustiquaires imprégnées, qui ne concernent que 10% des ménages [59], bien en dessous des objectifs fixés par les programmes de contrôle. Le diagnostic du paludisme à Djibouti repose principalement sur les tests rapides (TDR) détectant la protéine riche en histidine 2 (HRP2) spécifique à *P. falciparum.* Cependant, des études ont révélé que 0,6% de ces tests donnent des résultats faussement négatifs en raison de délétions dans les gènes pfhrp2/pfhrp3 [33,66]. La microscopie par examen de goutte épaisse reste la méthode de référence [69], tandis que la PCR est utilisée pour les confirmations complexes. Face à ces limitations, l’OMS recommande désormais l’utilisation de TDR combinant la détection de HRP2 et de lactate déshydrogénase dans les zones à risque de faux négatifs [33], une approche particulièrement pertinente dans le contexte djiboutien.

Le spectre clinique du paludisme à Djibouti varie selon l’espèce plasmodiale en cause. *P. falciparum* est responsable des formes les plus sévères, incluant neuropaludisme, anémie sévère posttraitement [9] et splénomégalie [15]. *P. vivax* et *P. ovale* provoquent principalement des accès fébriles et des récurrences, mais peuvent aussi entraîner des complications atypiques comme des péricardites [19]. Des perturbations hématologiques, notamment des thrombocytoses, ont été régulièrement observées [55]. Les populations les plus vulnérables incluent les jeunes enfants (avec une prévalence rurale de 0% chez les 0,5-4 ans) [61] et les militaires en poste dans le pays [72], deux groupes nécessitant une attention particulière dans les stratégies de prévention et de prise en charge.

La prise en charge thérapeutique diffère selon l’espèce plasmodiale. Pour *P. falciparum,* les combinaisons thérapeutiques à base d’artémisinine (ACT) constituent le traitement de référence, bien que des échecs aient été documentés avec la dihydroartémisinine-pipéraquine [72,80]. La tafénoquine a démontré une efficacité intéressante dans ce contexte [73]. Le traitement de *P. vivax* associe classiquement chloroquine et primaquine. Cependant, la résistance à la chloroquine, observée de manière significative depuis les épidémies de 1988-1989 [30,69], reste une préoccupation majeure pour les cliniciens. Ces résistances multiples compromettent sérieusement les objectifs nationaux ambitieux visant l’élimination du paludisme d’ici 2030 [90].

Malgré les efforts, une recrudescence de *P. falciparum* et *P. vivax* est anticipée [83,84,86]. Les défis majeurs incluent la résistance vectorielle, les limites diagnostiques et les importations transfrontalières. Le renforcement de la surveillance et l’innovation thérapeutique sont essentiels pour atteindre les objectifs d’élimination [90].

### La filariose lymphatique

La filariose lymphatique reste l’une des maladies les plus rares touchant la population djiboutienne, les données disponibles étant contradictoires et ne permettant pas de statuer clairement sur sa prévalence.

Initialement, la République de Djibouti était classée parmi les pays ayant des antécédents de transmission de la filariose lymphatique. Entre 2000 et 2001, des enquêtes communautaires à l’échelle nationale ont confirmé ce statut grâce à des tests de dépistage par bandelette dans l’ensemble des zones potentiellement endémiques [63]. Par la suite, des activités de cartographie ont été prévues pour évaluer la situation [21]. Cependant, une décennie plus tard, la possibilité d’une transmission active en République de Djibouti reste incertaine, faute d’enquêtes épidémiologiques récentes. Des cas importés depuis le Yémen, où la maladie est endémique, sont suspectés [65]. La proximité géographique et l’afflux important de migrants fuyant la guerre accentuent ce risque. Entre 2016 et 2017, une nouvelle enquête utilisant des bandelettes de dépistage a révélé une prévalence de 0,3% (ministère de la Santé, données non publiées) [91].

### La dengue

Bien que moins documentée que le paludisme, la dengue est mentionnée comme une menace émergente. Le principal vecteur est *Aedes aegypti.* Entre 1987 et 1988, aucun cas dû au virus de la dengue n’a été détecté à Djibouti [81]. Cependant, une épidémie majeure de dengue de sérotype 2 (DENV-2) a frappé le pays d’octobre 1991 à février 1992, avec environ 12 000 cas estimés et un taux d’attaque de 3,8% confirmé par des analyses sérologiques. Cette épidémie suggère une introduction récente du DENV-2 dans la région, hypothèse soutenue par plusieurs études [32,77]. Entre 1996 et 1999, le DENV-2 a continué de circuler silencieusement, sans déclencher d’épidémie [5,53]. Une enquête sérologique menée durant cette période s’est par ailleurs révélée négative pour les sérotypes DENV-1 et DENV-4 [77].

En 2000, un cas de dengue compliquée d’une crise maniaque a été signalé chez un militaire de retour de Djibouti [74]. Parallèlement, des travaux génomiques ont permis de décrire entièrement la souche DENV-1 circulant dans le pays. L’analyse phylogénétique indique que cette souche djiboutienne serait le progéniteur africain de la souche cambodgienne et le parent principal de celle identifiée à Singapour [89]. La souche DENV-2 serait apparentée avec celles du Kenya, de l’île de la Réunion et de Guangzhou en Chine [36]. La dengue est aujourd’hui considérée comme une maladie endémique à Djibouti [64,83]. Le sérotype DENV-1 a été identifié à plusieurs reprises, notamment chez *Ae. aegypti* [50]. Sa circulation a été documentée dans des études épidémiologiques [43,44]. Entre 2010 et 2011, une surveillance renforcée au sein de l’armée française stationnée à Djibouti a révélé pour la première fois la cocirculation des DENV-1 et DENV-3 [42,44]. Une étude sérologique rapporte une séroprévalence de 21,8% chez les adultes, nettement supérieure à celle d’autres arbovirus [6]. Contrairement à d’autres régions, elle n’affecte pas majoritairement les enfants.

Entre 2019 et 2020, une co-circulation d’arbovirus incluant des cas de dengue et de chikungunya ainsi que de paludisme a été observée [34]. La transmission est annuelle, avec un pic de mars à octobre, et le risque est jugé élevé. En l’absence de mesures de protection adaptées pour les personnes exposées, le taux d’infection mensuel est estimé entre 1% et 50% parmi les populations à risque [88].

### Le chikungunya

La première enquête sérologique humaine, réalisée en 1987, a révélé que le virus du chikungunya (CHIKV) n’était qu’une faible menace pour la population. Un seul sujet séropositif, originaire d’Éthiopie, présentait des anticorps contre le CHIKV, probablement développés lors d’une infection antérieure [81]. Jusqu’en 2009, seuls quelques cas isolés de transmission autochtone avaient été identifiés sur le territoire [3]. Une enquête menée en 2010 a montré que seulement 2,6% de la population possédaient des anticorps contre ce virus. Cependant, une épidémie de CHIKV a été signalée peu après, en 2011 [6].

En 2017, la souche indienne du virus a été détectée chez un Français résidant à Djibouti, diagnostiqué avec une infection aiguë autochtone. Cette observation a suggéré une possible implication de cette souche dans une épidémie dans la Corne de l’Afrique. Le virus est réapparu en novembre 2019. L’analyse phylogénétique a confirmé que la souche djiboutienne appartenait à la lignée indienne du génotype ECSA (Afrique de l’Est/ Centrale/Sud). Deux mutations, augmentant significativement l’adaptabilité du virus à *Ae. aegypti* seul vecteur présent dans la ville de Djibouti, ont été identifiées [27].

Entre 2019 et 2020, une épidémie de chikungunya a touché la ville de Djibouti, coïncidant avec une circulation simultanée du virus de la dengue et du paludisme. Fait notable, des infections secondaires (pneumonie, gingivite avec candidose buccale) ont été observées chez quatre patients atteints de chikungunya [34]. De plus, un cas chez des membres des forces armées américaines a dû être hospitalisé pendant plus de 12 mois [87].

*Ae. aegypti,* l’espèce responsable de la transmission du chikungunya et de la dengue [45] et [75], a été détectée dans le pays en 1976 [76]. La transmission du chikungunya est possible tout au long de l’année, avec un pic entre mars et octobre, et un risque considéré comme intermédiaire [88].

### La fièvre de la vallée du Rift

Jusqu’en 1987, aucune infection humaine par le virus de la fièvre de la vallée du Rift (RVF) n’a été recensée en République de Djibouti [81]. Par la suite, une étude sérologique menée auprès de travailleurs d’abattoirs n’a détecté aucune séropositivité pour le RVF [14]. Une étude réalisée sur des sérums de dromadaires provenant de Djibouti, a confirmé l’absence d’anticorps contre ce virus [58].

Dans les années 2000, une épidémie de RVF aux conséquences catastrophiques pour le bétail a frappé la Corne de l’Afrique, incluant potentiellement Djibouti [3]. Toutefois, aucun cas n’a été explicitement signalé dans le pays. En 2021, une étude pilote a évalué une méthode innovante de détection des pathogènes vectoriels, combinant des cartes de détection *Fast Technology for Analysis of Nucleic Acids* (FTA^®^) et une PCR quantitative en temps réel. Cette approche n’a révélé aucune trace du RVF parmi les cibles analysées [51].

En 2024, aucun foyer épidémique de RVF n’a été officiellement enregistré à Djibouti, malgré une surveillance active. L’absence récurrente de cas confirmés, tant chez l’humain que chez l’animal, suggère que le territoire est indemne de cette arbovirose, une situation particulière pour la Corne de l’Afrique, région endémique.

### La fièvre du Nil occidental

Les premières circulations autochtones du virus du Nil occidental (VNO) ont été détectées chez des chevaux entre juillet 2004 et août 2005, avec un taux de séroconversion de 9% [12]. Une épidémie majeure a ensuite touché la population humaine de la région rurale de Djibouti en 2006, avec un taux d’attaque moyen de 16% [3].

Plusieurs cas cliniques d’infection par le VNO ont été documentés parmi les militaires français stationnés à Djibouti: trois cas en 2014, un en 2015 et un début 2016, confirmant une circulation virale continue mais variable selon les années [52]. Les analyses ont révélé que la lignée 2 du VNO était responsable des cas identifiés entre le 20 décembre 2010 et le 7 janvier 2011 [26]. Fait notable, le VNO a été impliqué dans un cas de paralysie flasque aiguë chez un patient ayant séjourné à Djibouti, initialement suspecté de poliomyélite [47].

Deux espèces de moustiques assurent la transmission du VNO: *Culex quinquefasciatus* (signalé dès 1979), actif en zones péri-urbaines et rurales [30] et *Cx. pipiens torridus* (détecté en 2009), principalement urbain [24,26]. La transmission persiste toute l’année, avec un pic d’activité de mars à octobre, et le risque global est classé comme intermédiaire [88]. Une étude a confirmé la présence du VNO chez des vecteurs, sans toutefois préciser la lignée virale [50].

### La fièvre jaune

Initialement, la République de Djibouti avait été classée comme zone d’endémicité amarile par l’UNRRA (Administration des Nations Unies pour le secours et la réhabilitation). Cette classification était conditionnelle. L’exclusion de l’endémicité était permise à condition que l’indice stégo-myien de Breteau (pour *Ae. aegypti)* dans le port de Djibouti reste inférieur à 1% et qu’un rapport trimestriel sur cet indice soit transmis à l’OMS [62].

Les données disponibles suggèrent une absence historique de fièvre jaune sur le territoire. En 1947, une enquête sérologique menée à Djibouti et Tadjourah concluait à une faible probabilité d’épidémie récente, malgré un échantillonnage limité [8]; en 1987 une étude utilisant des tests d’immunofluorescence indirecte n’a détecté aucune infection humaine récente par le virus amaril [81].

Certaines sources affirment catégoriquement qu’aucun cas n’a jamais été rapporté [56], ce qui corrobore le statut officiel de « pays sans risque » dans la classification de l’OMS [35]. Bien qu’indemne historiquement, Djibouti présente des facteurs de risque. La zone portuaire apparaît comme la plus exposée à une introduction virale. La présence d’Ae. *aegypti* est confirmée depuis 1976 [76]. Une surveillance entomologique doit être maintenue (conditions UNRRA). Cette situation justifie une vigilance particulière malgré l’absence de circulation avérée [29].

### Le Zika

La République de Djibouti n’a pas enregistré de voyageur en provenance de zones où le virus Zika était endémique. Une exception notable est toutefois survenue lors des Jeux Olympiques de Rio de Janeiro en 2016, où une délégation djiboutienne séjourna [31]. Bien que ce retour ait pu représenter un risque d’importation du virus, aucun cas n’a été signalé confirmant ainsi l’absence du Zika sur le territoire.

## Discussion

Les maladies vectorielles à transmission culicidienne à Djibouti ont suscité un intérêt croissant au cours de la dernière décennie. Cette attention accrue s’explique par une double dynamique: l’émergence récente d’une espèce vectorielle invasive *(An. stephensi)* et une recrudescence préoccupante de pathologies endémiques, exacerbée par les changements climatiques et l’urbanisation rapide.

Les résultats de notre travail révèlent qu’au moins quatre maladies transmises par les moustiques ont été identifiées avec certitude, dont deux persistent de manière continue tout au long de l’année: le paludisme et la dengue. Les deux autres, la fièvre du Nil occidental et le chikungunya, n’ont été signalées que ponctuellement.

### Surveillance des maladies à Djibouti

La surveillance épidémiologique repose sur la collecte, l’analyse et l’interprétation continues de données sanitaires, fournissant des informations essentielles pour la planification, la mise en œuvre et l’évaluation des politiques de santé publique. À Djibouti, les maladies à déclaration obligatoire incluent les fièvres hémorragiques (dont la fièvre de la vallée du Rift), le MERS-Coronavirus (syndrome respiratoire aigu sévère du Moyen Orient), la brucellose et la rage, soit quatre zoonoses majeures.

Les professionnels de santé (médecins et vétérinaires) doivent également signaler d’autres pathologies à haut risque épidémique, comme le virus du Nil occidental, la dengue et la fièvre jaune [68]. Conformément au Règlement sanitaire international de l’OMS, les États membres doivent notifier tout événement susceptible de constituer une urgence sanitaire mondiale [67]. Une communication rapide des flambées épidémiques est cruciale pour alerter les acteurs de la santé et renforcer la vigilance, notamment vis-à-vis des patients revenant de zones endémiques.

Le risque d’émergence de maladies depuis l’Éthiopie existe. Les migrations entre Djibouti et l’Éthiopie, où des maladies comme la fièvre jaune [22], le Zika [71], la filariose lymphatique [20] ou la fièvre de la vallée du Rift [60] sont présentes, constituent un facteur de risque critique: par exemple, la dissémination des sérotypes de la dengue via les voyageurs est bien documentée, comme en témoigne l’introduction du DENV-3 en Amérique latine dans les années 1994 à partir de l’Asie [13]. Concernant le paludisme, cinq espèces du genre *Plasmodium* sont responsables de la maladie chez l’humain: *P. falciparum* [48], *P. knowlesi* [84], *P. malariae* [18], *P. ovale* [17] et *P. vivax* [16]. De plus, il convient de souligner que *P. ovale* a été principalement observé chez des militaires français en provenance d’Afrique de l’Ouest, où cette espèce est endémique [46]. Cela suggère une origine exogène à Djibouti. Néanmoins, ce parasite pourrait se propager localement via la piqûre d’un patient infecté par *An. stephensi* [25], un vecteur compétent capable de favoriser sa circulation [16].

Les réservoirs animaux et humains jouent un rôle clé dans la persistance des maladies. Comme pour le paludisme, les porteurs asymptomatiques de la dengue pourraient maintenir une transmission silencieuse, un phénomène décrit dans des contextes de faible surveillance [82]. Le virus du Nil occidental, absent à Djibouti mais présent en Éthiopie, pourrait être transporté par des oiseaux migrateurs comme ceux impliqués dans la diffusion du virus en Afrique, en Europe et en Amérique [41,49].

On suggère de renforcer la surveillance syndromique avec l’intégration des tests moléculaires (RT-PCR) et sérologiques pour détecter les coinfections et les cas asymptomatiques des virus [4] ainsi que l’investigation des réservoirs animaux et des dynamiques vectorielles, en s’inspirant des travaux réalisés en Afrique centrale [40].

### Lutte contre les maladies vectorielles

La gestion intégrée des vecteurs est un pilier central de cette lutte. L’Institut national de santé publique et le Programme national de lutte contre le paludisme (PNLP) surveillent activement les moustiques vecteurs présents à Djibouti, tels que *An. gambiae* et *An. stephensi* et *Ae. aegypti.*

La problématique a fait l’objet de nombreuses investigations scientifiques, notamment dans l’étude pionnière de Abdi Khaireh Bouh [37] qui porte sur le paludisme à Djibouti en vue d’une pré-élimination. Ce travail, centré sur les spécificités d’un contexte de transmission résiduelle, a analysé les défis posés par la persistance de foyers endémiques localisés et l’impact des flux migratoires transfrontaliers sur les risques récurrents d’importation parasitaire. L’auteur souligne notamment la vulnérabilité du territoire djiboutien, situé dans un corridor épidémiologique où la mobilité des populations en provenance de zones hyper-endémiques (Éthiopie, Somalie) complexifie les stratégies de contrôle. Cette recherche a également mis en lumière l’hétérogénéité spatiotemporelle des cas autochtones, plaidant pour une approche différentielle combinant surveillance moléculaire et renforcement des barrières prophylactiques aux points d’entrée.

Depuis 2024, un projet de collaboration a été lancé entre la biotech Oxitec, spécialisée dans les solutions de lutte anti-vectorielle par modification génétique, l’ONG Association Mutualis et le PNLP [70]. Cette initiative vise à endiguer la recrudescence du paludisme liée à l’expansion *d’An. stephensi,* une espèce invasive particulièrement résistante aux méthodes de contrôle traditionnelles. Ce projet consiste en des lâchers de mâles de cette espèce portant un gène létal pour les femelles et ce pour réduire de 90% la population.

D’autres méthodes sont utilisées, notamment la pulvérisation d’insecticides de troisième génération, les moustiquaires imprégnées et la surveillance des gîtes larvaires. Cependant, il y a des difficultés liées à la résistance aux insecticides et à l’urbanisation anarchique.

### Sensibilisation et prévention

Des campagnes d’éducation sanitaire sur les maladies vectorielles et les méthodes de lutte anti-vectorielle sont diffusées via des programmes télévisés et radiophoniques et des annonces publicitaires ciblées. Ces actions visent à améliorer les connaissances du public et à promouvoir des comportements préventifs.

Pour renforcer l’impact des campagnes de sensibilisation sur les maladies vectorielles et sur les méthodes de lutte anti-vectorielle, une approche multicanale, inclusive et adaptée aux contextes locaux est essentielle. Nous proposons:

- la diversification des canaux de communication, notamment l’utilisation des réseaux sociaux (Facebook, X) pour toucher les jeunes avec des contenus interactifs (vidéos courtes, infographies, témoignages), et le développement d’applications mobiles éducatives avec des alertes en temps réel sur les périodes à risque ou les épidémies;
- l’organisation d’ateliers dans les écoles, les marchés et les centres de santé, animés par des agents locaux ou des influenceurs communautaires et la distribution des supports visuels (affiches, dépliants) en langues locales, illustrant les gestes préventifs (utilisation de moustiquaires, élimination des eaux stagnantes);
- l’adaptation du contenu aux publics spécifiques en mettant l’accent sur l’assainissement de l’environnement et les méthodes de protection traditionnelles (moustiquaires imprégnées) aussi bien en zones rurales qu’en zones urbaines et en se focalisant sur la prévention individuelle (répulsifs, vêtements couvrants) et la gestion des déchets en zone urbaine. Pour les voyageurs, il faudrait mettre en place des partenariats avec les agences de tourisme pour diffuser des conseils avant les déplacements et après;
- l’implication des chefs religieux, enseignants, personnalités médiatiques pour renforcer la crédibilité des messages.

## Conclusions

Notre étude sur les maladies transmises par les moustiques à Djibouti montre que le pays a historiquement été touché par quatre d’entre elles: le chikungunya, la dengue, la fièvre du Nil occidental et le paludisme. Ce dernier a connu une recrudescence marquée en raison de l’émergence de nouvelles espèces de vecteurs sur le territoire, contribuant ainsi à la persistance de sa transmission.

D’autres pathologies comme la fièvre de la vallée du Rift, la fièvre jaune, la filariose lymphatique et le Zika ont été associées à la République de Djibouti dans la littérature. Toutefois, les données disponibles indiquent qu’aucun cas autochtone ou importé de ces maladies n’a été confirmé à ce jour. Des recherches approfondies sont nécessaires pour caractériser les agents pathogènes et les vecteurs potentiels, ainsi que pour mieux comprendre les signes cliniques associés. Ces travaux permettraient d’éclairer des questions encore non résolues.

Djibouti fait face à une crise sanitaire complexe, marquée par l’émergence d’un vecteur invasif *An. stephensi* et la recrudescence du paludisme. Les stratégies innovantes, comme les moustiques génétiquement modifiés, coexistent avec des défis structurels (résistance aux insecticides, urbanisation). Une approche intégrée, associant surveillance épidémiologique dans un contexte de changement climatique, contrôle biologique et coopération régionale, sera cruciale pour contrer ces menaces sachant que 90% de la population djiboutienne vit en ville, augmentant ainsi l’exposition aux vecteurs urbains.

Il est à noter que *An. stephensi* est déjà signalé en Éthiopie, en Somalie et au Soudan, menaçant de propager le paludisme urbain en Afrique de l’Est. Une collaboration renforcée entre l’OMS, les autorités sanitaires, les professionnels de santé et la communauté est essentielle pour prévenir l’importation d’agents infectieux et limiter l’impact des infections contractées localement.

## Financement

Cette étude n’a reçu aucun financement.

## Contribution des auteurs

Abdoulgabar ABDOURAHMAN OMAR: prospection bibliographique, définition de la méthodologie, rédaction de l’article et validation. Oumnia HIMMI: conception de l’étude, correction et validation du manuscrit.

## Déclaration de liens d’intérêt

Les auteurs ne déclarent aucun lien d’intérêts.
