# Supplementary material for: Mosquito-borne diseases in the Republic of Djibouti: a review of the literature
Source: Med Trop Sante Int. 2025 Nov 10;5(4):mtsi.v5i4.2025.778. [Article in French] doi: 10.48327/mtsi.v5i4.2025.778 (PMC12957994; doi:10.48327/mtsi.v5i4.2025.778)
Supplement: Related texts

# Research article: Mosquito-borne diseases in the Republic of Djibouti: a review of the literature

## Introduction

Mosquitoes are known for their ability to transmit pathogens, primarily parasites and viruses. They are responsible for transmitting diseases to humans and animals [23]. A recent literature review [2] mentions 37 species of mosquitoes in Djibouti, eight of which are potential disease vectors, including malaria, lymphatic filariasis, dengue fever, yellow fever, West Nile virus, and chikungunya. Notably, *Anopheles stephensi* was introduced in 2012, coinciding with an increase in malaria cases in the country [25].

The Republic of Djibouti is a former French colony located in the Horn of Africa. It is bordered by Eritrea to the north, Ethiopia to the west, Somalia to the south, and the Red Sea to the east (Fig. 1). This work is part of a doctoral research project on mosquito ecology in Djibouti and follows the publication of a literature review on Culicidae in the country [2]. The objective of this study is to provide a clear and comprehensive summary of mosquito-borne diseases and associated pathogens based on reference articles.

**Figure 1:** Map of the Republic of Djibouti: (a) location in Africa, (b) neighboring countries and (c) regions

## Materials and methods

In accordance with the PRISMA (Preferred Reporting Items for Systematic Reviews and Meta-Analyses) guidelines, a review of the published literature on mosquito-borne diseases in Djibouti was conducted. The databases consulted were PubMed, ScienceDirect, Google Scholar, and Springer Nature. To maximize comprehensiveness and limit selection bias, keywords in English and French covering the main vector-borne diseases of concern were used. The search terms used were as follows:

- *“Moustiques de Djibouti”* / *“Djibouti mosquitoes”;*
- *“Maladies vectorielles à Djibouti”* / *“Vector-borne diseases in Djibouti”;*
- *“Dengue Djibouti”;*
- *“Fièvre jaune Djibouti »* / « *Yellow Fever Djibouti”*;
- « *Fièvre de la vallée du Rift Djibouti* » / « *Rift Valley Fever Djibouti”;*
- *“Filariose lymphatique Djibouti”*/ *“Lymphatic filariasis Djibouti”;*
- *“Virus du Nil occidental Djibouti »»* / « *West Nile virus Djibouti*”;
- *“Paludisme Djibouti* » / « *Malaria Djibouti”;*
- *“Zika Djibouti”;*
- *“Encéphalite japonaise Djibouti”/ “Japanese encephalitis Djibouti*”

The studies included in this review are original research studies. They describe results related to mosquito-borne diseases in the Republic of Djibouti, focusing on molecular and genetic diversity, as well as human and animal risk factors associated with ecological and climatic conditions. The studies also describe spatio-temporal and predictive risk modeling.

We excluded articles that mentioned mosquito-borne diseases but focused more on purely disciplinary studies related to microbiology, entomology, and hematology, as well as review articles, as illustrated in Figure 2.

**Figure 2:** Organizational chart for the selection of articles for a systematic review of mosquito-borne diseases in Djibouti

## Results

A total of 193 articles were identified in this systematic review of the literature on mosquito-borne diseases in the Republic of Djibouti. After eliminating duplicates and irrelevant articles, 82 references were selected for analysis (Fig. 2).

This article summarizes the main arboviral and parasitic diseases of major epidemiological importance in Djibouti.

### Malaria

Djibouti has unstable malaria transmission [1,64]. After the first cases were reported in 1901, the disease became endemic in 1991 [10,11,57], reaching peaks of 7,000 cases per year [54,78]. Two urban epidemics in 2013-2014 led to 70,000 cases in 2020 and were linked to *Anopheles stephensi* [25,85,92]. Analysis of confirmed cases reveals a clear predominance of *Plasmodium falciparum*, which is responsible for approximately 90% of infections [39]. Recent data (2018-2021) confirm this trend, attributing 83% of cases to this species [59]. *Plasmodium vivax* accounts for 5-10% of overall cases [39], with 11% during the 2018-2021 period [59]. *Plasmodium ovale* remains marginal, accounting for only 3% of infections [72]. Transmission persists throughout the year, but it is highly seasonal with an increase between November and May [88]. Genetic studies show a change in parasite diversity. It was high before 1999 [79], and then declined thereafter [7], probably reflecting changes in transmission dynamics and the influence of parasite imports from neighboring Ethiopia [28,38].

The arrival of *Anopheles stephensi* in 2012 profoundly altered the malaria epidemiology in Djibouti [25,85]. This vector exhibits several concerning characteristics, including resistance to the four main classes of insecticides [85], excellent adaptation to urban environments, and pronounced endophilic behavior. The traditional vectors, *Anopheles gambiae* and *Anopheles arabiensis,* now play a secondary role [92]. Efforts to combat these vectors are hindered by the low usage of impregnated mosquito nets, which are employed in only 10% of households, far below the targets established by control programs [59]. In Djibouti, malaria diagnosis relies mainly on rapid diagnostic tests (RDTs) that detect histidine-rich protein 2 (HRP2), which is specific to *P. falciparum.* However, studies have shown that 0.6% of these tests yield false-negative results due to deletions in the pfhrp2/pfhrp3 genes [33,66]. Thick smear microscopy remains the gold standard [69], while PCR is used for complex confirmations. Due to these limitations, the WHO now recommends using RDTs that combine HRP2 and lactate dehydrogenase detection in areas at risk of false negatives [33], an approach that is particularly relevant in the context of Djibouti. The clinical spectrum of malaria in Djibouti varies depending on the species of *Plasmodium* involved. *P. falciparum* causes the most severe forms, including neuropaludism, severe post-treatment anemia [9] and splenomegaly [15]. *P. vivax* and *P. ovale* primarily cause fever and recurrence, though they can also lead to atypical complications, such as pericarditis [19]. Hematological disorders, particularly thrombocytosis, are regularly observed [55]. The most vulnerable populations include young children, with a rural prevalence of 0% among 0.5-4-year-olds [61] and military personnel stationed in the country [72], two groups requiring special attention in prevention and management strategies.

Therapeutic management differs depending on the *Plasmodium* species. For *P. falciparum,* artemisinin-based combination therapies (ACTs) are the standard treatment, though dihydroar-temisinin-piperaquine has been associated with treatment failure [72,80]. Tafenoquine has shown promising efficacy in this context [73]. Treatment for *P. vivax* typically combines chloroquine and primaquine. However, chloroquine resistance, which has been observed since the 1988-1989 epidemics, remains a major concern for clinicians [30,69]. These multiple resistances seriously compromise national goals to eliminate malaria by 2030 [90].

Despite these efforts, a resurgence of *P. falciparum* and *P. vivax* is anticipated [83,84,86]. Major challenges include vector resistance, diagnostic limitations, and cross-border importation of the parasite. Strengthened surveillance and therapeutic innovation are essential to achieving these goals [90].

### Lymphatic Filariasis

Lymphatic filariasis is one of the rarest diseases affecting the population of Djibouti. Available data is contradictory and does not allow for a clear determination of its prevalence.

Initially, the Republic of Djibouti was classified as a country with a history of lymphatic filariasis transmission. Between 2000 and 2001, nationwide community surveys confirmed this status through strip testing in all potentially endemic areas [63]. Subsequently, mapping activities were planned to assess the situation [21]. However, due to a lack of recent epidemiological surveys, the possibility of active transmission in the Republic of Djibouti remains uncertain a decade later. Cases imported from Yemen, where the disease is endemic, are suspected [65]. The geographical proximity and the large influx of migrants fleeing the war increase this risk. Between 2016 and 2017, a new survey using screening strips revealed a prevalence of 0.3% (Ministry of Health, unpublished data) [91].

### Dengue

Although less well documented than malaria, dengue is mentioned as an emerging threat. The main vector is *Aedes aegypti.*

Between 1987 and 1988, no dengue virus cases were detected in Djibouti [81]. However, the country experienced a major epidemic of dengue fever serotype 2 (DENV-2) from October 1991 to February 1992, with an estimated 12,000 cases and an attack rate of 3.8%, as confirmed by serological analyses. This epidemic suggests a recent introduction of DENV-2 into the region, a hypothesis supported by several studies [32,77]. DENV-2 continued to circulate silently between 1996 and 1999, without triggering an epidemic [5,53]. A serological survey conducted during this period was also negative for the DENV-1 and DENV-4 serotypes [77].

In 2000, a case of dengue fever complicated by a manic episode was reported in a soldier who had returned from Djibouti [74]. Around that time, genomic research allowed for a complete description of the DENV-1 strain circulating in the country. Phylogenetic analysis indicates that the Djiboutian strain is the progenitor of the Cambodian strain and the parent strain of the one identified in Singapore [89]. The DENV-2 strain is believed to be related to strains found in Kenya, Réunion Island, and Guangzhou, China [36] Dengue is now considered endemic in Djibouti [64,83]. The DENV-1 serotype has been identified several times, particularly in *Ae. aegypti* [50]. Its circulation has been documented in epidemiological studies [43,44]. Between 2010 and 2011, enhanced surveillance by the French military in Djibouti revealed the co-circulation of DENV-1 and DENV-3 for the first time [42,44]. A serological study reported a seroprevalence of 21.8% in adults, which is significantly higher than that of other arboviruses [6]. Unlike in other regions, dengue does not primarily affect children.

From 2019 to 2020, the co-circulation of arboviruses, including dengue, chikungunya, and malaria, was observed [34]. Transmission occurs annually, peaking from March to October, and the risk is considered high. Without appropriate protective measures for exposed individuals, the estimated monthly infection rate among at-risk populations is between 1% and 50% [88].

### Chikungunya

The first human serological survey, conducted in 1987, revealed that the chikungunya virus (CHIKV) posed a low threat to the population. Only one seropositive individual, who was originally from Ethiopia, had antibodies against CHIKV, which were likely developed during a previous infection [81]. Until 2009, only a few cases of local transmission were identified in the territory [3]. A 2010 survey showed that only 2.6% of the population had antibodies against the virus. However, a CHIKV epidemic was reported shortly thereafter in 2011 [6].

In 2017, the Indian strain of the virus was detected in a French citizen residing in Djibouti who was diagnosed with an acute indigenous infection. This observation suggested possible involvement of this strain in an epidemic in the Horn of Africa. The virus reappeared in November 2019. Phylogenetic analysis confirmed that the Djiboutian strain belonged to the Indian lineage of the ECSA (East/Central/South Africa) genotype. Two mutations that significantly increased the virus’s adaptability to *Ae. aegypti,* the only vector present in Djibouti, were identified [27]. Between 2019 and 2020, a chikungunya epidemic affected the city of Djibouti, coinciding with the circulation of dengue virus and malaria. Secondary infections, including pneumonia and gingivitis with oral candidiasis, were observed in four patients with chikungunya [34]. Additionally, one case among members of the U.S. Armed Forces required hospitalization for over 12 months [87]. *Ae. aegypti,* the species responsible for transmitting chikungunya and dengue [45] and [75], was first detected in the country in 1976 [76]. Chikungunya transmission is possible year-round, peaking between March and October, with an intermediate risk [88].

### Rift Valley Fever

Until 1987, there had been no reported cases of human infection with the Rift Valley fever virus (RVF) in the Republic of Djibouti [81]. A subsequent serological study conducted among slaughterhouse workers detected no seropositivity for RVF [14]. A study of camel serum from Djibouti confirmed the absence of antibodies against the virus [58].

In the 2000s, a catastrophic RVF epidemic struck the Horn of Africa, potentially including Djibouti [3]. However, no cases were explicitly reported in the country. In 2021, a pilot study evaluated an innovative method for detecting vector pathogens. This method combined Fast Technology for Analysis of Nucleic Acids (FTA^®^) indicator cards and quantitative real-time PCR. This approach revealed no trace of RVF among the analyzed samples [51].

As of 2024, there have been no official reports of RVF epidemic outbreaks in Djibouti, despite active surveillance. The recurring absence of confirmed cases in both humans and animals suggests that the territory is free of this arbovirus, a unique situation for the Horn of Africa, an endemic region.

### West Nile fever

The first indigenous circulation of the West Nile virus (WNV) was detected in horses between July 2004 and August 2005, with a seroconversion rate of 9% [12]. A major epidemic then affected the human population in rural Djibouti in 2006, with an average attack rate of 16% [3].

Several cases of West Nile virus (WNV) infection have been documented among French military personnel stationed in Djibouti: three cases in 2014, one case in 2015, and one case in early 2016. These cases confirm continuous, though variable, viral circulation over the years [52].

Analyses revealed that WNV lineage 2 was responsible for the cases identified between December 20, 2010, and January 7, 2011 [26]. Notably, WNV was implicated in a case of acute flaccid paralysis in a patient who had stayed in Djibouti and was initially suspected of having poliomyelitis [47].

Two species of mosquitoes transmit WNV: *Culex quinquefasciatus*, active in peri-urban and rural areas [30] and reported since 1979, and *Cx. pipiens torridus,* mainly urban and detected in 2009 [24,26]. Transmission persists throughout the year, with peak activity from March to October, and the overall risk is classified as intermediate [88]. One study confirmed the presence of WNV in vectors but did not specify the viral lineage [50].

### Yellow fever

Initially, the Republic of Djibouti was classified as an Amarile endemic area by the United Nations Relief and Rehabilitation Administration (UNRRA). This classification was conditional. The country could be excluded from the classification if the Breteau stegomyian index (for *Ae. aegypti)* in the port of Djibouti remained below 1%, and if a quarterly report on this index was submitted to the WHO [62].

Available data suggest an historical absence of yellow fever in the territory. In 1947, a serological survey in Djibouti and Tadjourah concluded that the probability of a recent epidemic was low, despite limited sampling [8]. In 1987, an indirect immunofluorescence test study detected no recent human infections with the Amarile virus [81]. Some sources state categorically that no cases have ever been reported [56], which corroborates Djibouti’s official status as a “risk-free country” in the WHO classification [35]. Although Djibouti has historically been free of the virus, it presents risk factors. The port area is the most exposed to the introduction of the virus. The presence of *Ae. aegypti* has been confirmed since 1976 [76]. Entomological surveillance must be maintained (UNRRA conditions). Despite the absence of proven circulation, this situation warrants particular vigilance [29].

### Zika

The Republic of Djibouti has not recorded any travelers from areas where the Zika virus was endemic. However, there was one notable exception during the 2016 Olympic Games in Rio de Janeiro, when a delegation from Djibouti stayed there [31]. While this could have posed a risk of introducing the virus, no cases were reported, confirming the absence of Zika in the territory.

## Discussion

Culicid-transmitted vector-borne diseases in Djibouti have attracted growing interest over the last decade. This increased attention can be explained by two factors: the recent emergence of an invasive vector species *(An. stephensi)* and the resurgence of endemic diseases, which is exacerbated by climate change and rapid urbanization. Our work reveals that at least four mosquito-borne diseases have been identified, two of which persist throughout the year: malaria and dengue fever. The other two, West Nile fever and chikungunya, have only been reported sporadically.

### Disease surveillance in Djibouti

Epidemiological surveillance involves the ongoing collection, analysis, and interpretation of health data. This process provides essential information for planning, implementing, and evaluating public health policies. In Djibouti, notifiable diseases include hemorrhagic fevers (such as Rift Valley fever), Middle East Respiratory Syndrome, brucellosis, and rabies—four major zoonotic diseases. Health professionals, including doctors and veterinarians, must also report other diseases with a high epidemic risk, such as West Nile virus, dengue, and yellow fever [68].

According to the WHO International Health Regulations, member states must report any event that could constitute a global health emergency [67]. Rapid communication of outbreaks is crucial to alerting health professionals and increasing vigilance, particularly regarding patients returning from endemic areas.

There is a risk of diseases emerging from Ethiopia. Migration between Djibouti and Ethiopia, where diseases such as yellow fever [22], Zika [71], lymphatic filariasis [20], and Rift Valley fever [60] are present, is a critical risk factor. For example, the spread of dengue serotypes via travelers is well documented. This is evidenced by the introduction of DENV-3 into Latin America from Asia in 1994 [13]. Regarding malaria, five species of the genus *Plasmodium* cause the disease in humans: *P. falciparum* [48], *P. knowlesi* [84], *P. malariae* [18], *P. ovale* [17] and *P. vivax* [16]. Additionally, *P. ovale* has mainly been observed in French soldiers returning from West Africa, where it is endemic [46]. This suggests an exogenous origin in Djibouti. However, the parasite could spread locally through the bite of an infected *An. stephensi* [25], a competent vector capable of transmitting the parasite [16].

Animal and human reservoirs play a key role in the persistence of diseases. As with malaria, asymptomatic dengue carriers could maintain silent transmission, a phenomenon observed in areas with low surveillance [82]. Just as migratory birds spread the virus throughout Africa, Europe, and the Americas, they could also carry the West Nile virus. [41,49].

Strengthening syndromic surveillance by integrating molecular (RT-PCR) and serological tests could help detect co-infections and asymptomatic cases of viruses [4]. Additionally, investigating animal reservoirs and vector dynamics could provide insight, drawing inspiration from work carried out in Central Africa [40].

### Control of vector-borne diseases

Integrated vector management is central to this effort. The National Institute of Public Health and the National Malaria Control Program (PNLP) actively monitor mosquito vectors present in Djibouti, such as *An. gambiae, An. stephensi*, and *Ae. aegypti.*

This issue has been the subject of numerous scientific investigations, notably the pioneering study by Abdi Khaireh Bouh [37] on malaria in Djibouti prior to elimination. Focusing on the specifics of a residual transmission context, this work analyzed the challenges posed by the persistence of localized endemic foci and the impact of cross-border migration flows on the recurring risk of parasite importation. The author emphasizes Djibouti’s vulnerability, as it is situated in an epidemiological corridor where population mobility from hyper-endemic regions (e.g., Ethiopia and Somalia) complicates control strategies. The study also revealed the spatial and temporal heterogeneity of indigenous cases, suggesting a differential approach that combines molecular surveillance with the reinforcement of prophylactic barriers at entry points.

In 2024, Oxitec, a biotech company that specializes in vector control solutions through genetic modification, launched a collaborative project with the NGO Association Mutualis and the PNLP [70]. The project aims to stop the resurgence of malaria associated with the increase in *An. stephensi,* an invasive species that is resistant to traditional control methods. The project involves releasing male mosquitoes carrying a lethal gene to reduce the population by 90%.

Other methods are also being employed, including spraying third-generation insecticides, distributing impregnated mosquito nets, and monitoring larval breeding sites. However, difficulties related to insecticide resistance and uncontrolled urbanization remain.

### Awareness and prevention

Health education campaigns on vector-borne diseases and vector control methods are disseminated via television and radio programs, as well as targeted advertisements. These campaigns aim to improve public knowledge and promote preventive behaviors.

A multi-channel approach that is inclusive and adapted to local contexts is essential to strengthen the impact of awareness campaigns on vector-borne diseases and vector control methods. We propose:

- Diversify communication channels, particularly through social media (e.g., Facebook and X), to share interactive content (e.g., short videos, infographics, and testimonials) with young people and develop educational mobile apps with real-time alerts about periods of risk or epidemics.
- Organize workshops in schools, markets, and health centers led by local agents or community influencers. Distribute visual materials (e.g., posters and leaflets) in local languages that illustrate preventive measures (e.g., use of mosquito nets and elimination of standing water).
- Adapt the content to specific audiences by emphasizing. Emphasize environmental sanitation and traditional protection methods (e.g., impregnated mosquito nets) in rural and urban areas. Focus on individual prevention (e.g., repellents and protective clothing) and waste management in urban areas. For travelers, partnerships should be established with tourism agencies to disseminate advice before and after travel.
- Involve religious leaders, teachers, and media personalities to reinforce the credibility of the messages.

## Conclusions

Our study on mosquito-borne diseases in Djibouti shows that the country has historically experienced four of them: chikungunya, dengue, West Nile fever, and malaria. Malaria has seen a marked resurgence due to the emergence of new vector species in the territory, contributing to the persistence of its transmission.

The Republic of Djibouti has been associated with other diseases, such as Rift Valley fever, yellow fever, lymphatic filariasis, and Zika, in the literature. However, available data indicate that there have been no confirmed cases of these diseases, either indigenous or imported, to date.

Further research is needed to characterize potential pathogens and vectors and to better understand the associated clinical signs. This work would help shed light on unresolved issues. Djibouti is facing a complex health crisis marked by the emergence of the invasive mosquito species *An. stephensi* and the resurgence of malaria. Innovative strategies, such as the use of genetically modified mosquitoes, must coexist with structural challenges, such as insecticide resistance and urbanization. An integrated approach combining epidemiological surveillance, biological control, and regional cooperation will be crucial to counter these threats given that 90% of Djibouti’s population lives in cities, which increases exposure to urban vectors.

Notably, *An. stephensi* has already been reported in Ethiopia, Somalia, and Sudan, threatening to spread urban malaria in East Africa. Enhanced collaboration between the WHO, health authorities, health professionals, and the community is essential to prevent the importation of infectious agents and limit the impact of locally acquired infections.

## Funding

This study received no funding.

## Authors’ contributions:

Abdoulgabar ABDORAHMAN OMAR: bibliographic research, definition of methodology, writing of the article, and validation.

Oumnia HIMMI: study design, correction, and validation of the manuscript.

## Conflicts of interest

The authors declare no conflicts of interest.
